# Identification and targeting of an FGFR fusion in a pediatric thalamic “central oligodendroglioma”

**DOI:** 10.1038/s41698-017-0036-8

**Published:** 2017-09-07

**Authors:** Joseph R. Linzey, Bernard Marini, Kathryn McFadden, Adonis Lorenzana, Rajen Mody, Patricia L. Robertson, Carl Koschmann

**Affiliations:** 1 Department of Pediatrics, Division of Pediatric Hematology/Oncology, University of Michigan Medical School, Ann Arbor, MI 48109 USA; 2 Department of Pharmacy Services, University of Michigan Medical School, Ann Arbor, MI 48109 USA; 3 Department of Pathology, University of Michigan Medical School, Ann Arbor, MI 48109 USA; 4 Department of Pediatric Hematology-Oncology, St. John’s Hospital Grosse Pointe Woods, Detroit, MI 48236 USA; 5 Department of Pediatrics, Division of Neurology, University of Michigan Medical School, Ann Arbor, MI 48109 USA

## Abstract

Approximately 1–5% of pediatric intracranial tumors originate in the thalamus. While great strides have been made to identify consistent molecular markers in adult oligodendrogliomas, such as the 1p/19q co-deletion, it is widely recognized that pediatric oligodendrogliomas have a vastly different molecular make-up. While pediatric thalamic or “central oligodendrogliomas” are histologically similar to peripheral pediatric oligodendrogliomas, they are behaviorally distinct and likely represent a cohesive, but entirely different entity. We describe a case of a 10-year-old girl who was diagnosed with an anaplastic glioma with features consistent with the aggressive entity often diagnosed as central or thalamic oligodendroglioma. We performed whole-exome (paired tumor and germline DNA) and transcriptome (tumor RNA) sequencing, which demonstrated an *FGFR3-PHGDH* fusion. We describe this fusion and our rationale for pursuing personalized, targeted therapy for the patient’s tumor that may potentially play a role in the treatment of similar cases.

## Introduction

Tumors originating in the thalamus comprise ~1–5% of pediatric intracranial tumors.^1–3^ Pediatric thalamic tumors have a poor outcome and prognosis related to histologic type, histologic grade, and resectability.^3^ Prognosis is worse for tumors that are bithalamic compared to unilateral, high histologic grade, and possess *H3*.*3 K27M* mutations.^4–6^ This molecular and prognostic understanding primarily relates to astrocytic tumors, and little remains known about the molecular features of thalamic oligodendroglioma. Due to the essential functions of the thalamus and surrounding structures, as well as the difficult surgical approach to the midline, biopsy and resection were rare in the past. But with improved surgical techniques, total or partial resection is now more frequently performed.^7^

As the field of neuro-oncology became increasingly proficient in detecting molecular alterations within tumors, precision medicine for high-risk brain tumors has rapidly expanded in the clinic. Molecular analysis of adult gliomas has already begun to demonstrate specific molecular markers that designate prognostic and therapeutic benefit.^8, 9^ Unfortunately, it is also understood that while pediatric tumors may resemble adult tumors, they are often genetically dissimilar.^10^ Notably, while 50–70% of adult oligodendrogliomas demonstrate a 1p/19q co-deletion, this is only rarely seen in children with oligodendrogliomas.^8–11^ While pediatric thalamic or “central oligodendrogliomas” are histologically similar to peripheral oligodendrogliomas, they are behaviorally distinct and likely represent a cohesive, but entirely different entity.^3, 12–15^ Approximately 2/3 of central oligodendrogliomas are anaplastic (World Health Organization [WHO] grade III) at biopsy.

Overall, the biological basis for this behavior is not well understood and very little is known about the molecular attributes of pediatric central oligodendrogliomas. Here, we present a pediatric patient who was diagnosed with a thalamic anaplastic glioma with features consistent with the aggressive entity often diagnosed as central oligodendroglioma. Sequencing of the patient’s tumor led to the identification and therapeutic targeting of a fibroblast growth factor receptor (FGFR)-containing fusion.

## Case report

**Fig. 1:**
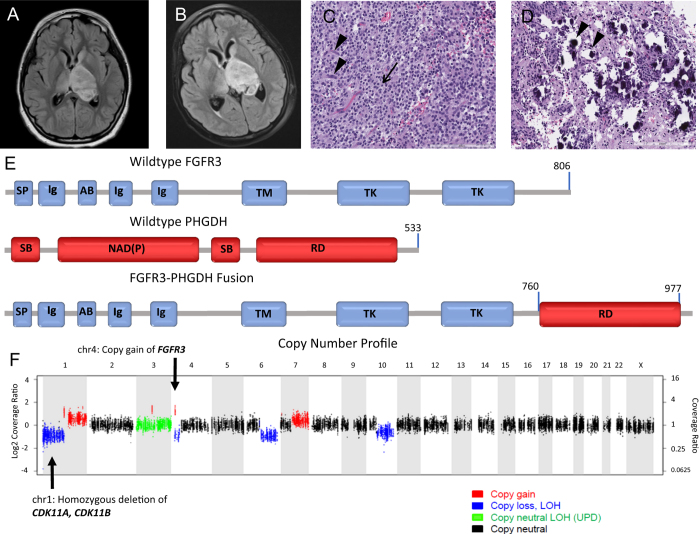
**a** Initial scan presenting axial T2 FLAIR image of the central tumor. Heterogenous, 5.1 × 3.8 × 3.4 cm ovoid mass with involvement of the left thalamus and internal capsule. Left lateral ventricle is distorted with displacement of the septum pellucidum and third ventricle. **b** Three-month follow-up axial T2 FLAIR image of central tumor status post treatment with chemotherapy. Concerning for tumor growth. **c** H&E-stained paraffin-embedded section showing moderately cellular glial neoplasm composed of relatively monomorphic round cells with clear cytoplasm and distinct cell borders (arrow), as well as arborizing capillaries (arrow heads). **d** From the same section as **c** showing calcospherites (arrow heads). **e** Fusion of FGFR3 with PHGDH. Top panel represents wildtype FGFR3. All exons are represented by blue or red squares while domains are represented by the gray bar (SP=signal peptide, Ig=immunoglobulin-like domain, AB=acid box, TM=transmembrane domain, TK=tyrosine kinase domain). The middle panel represents wildtype PHGDH (SB=substrate-binding domain, NAD(P)=NAD^+^/NADP^+^-binding domain, RD=regulatory domain). The lower panel represents the FGFT3-PHGDH fusion. Fusion occurs intracellularly, at the 3′ end of the FGFR3. FGFR3 exons 1~17 (p.D760) fused in-frame to PHGDH exons 9~12 (p.V316). The extracellular, transmembrane, and kinase domain for FGFR3 are all intact. Standard FGF inhibitors like ponatinib should theoretically work against this fusion assuming they have sufficient ability to cross the BBB. **f** Copy number profile of FGFR3-PHGDH fusion

A 10-year-old girl without a significant past medical history was presented, with a 3-week history of right-handed weakness and right facial drooping. A neurologic examination was significant with right-sided facial weakness and arm weakness. Magnetic resonance imaging (MRI) of the brain revealed a heterogeneously but minimally enhancing, 5.1 × 3.8 × 3.4 cm ovoid mass involving the left thalamus, upper brainstem, and internal capsule and extending posteriorly to the cerebellar peduncles. The left lateral ventricle appeared distorted with displacement of the septum pellucidum and third ventricle. Peripheral edema extended to the left caudate, bilateral hypothalami, bilateral temporal cortices, and right thalamus (Fig. 1a).

The patient underwent stereotactic needle biopsy of the mass, and pathology was initially suggestive of an anaplastic clear cell ependymoma. The patient was subsequently enrolled on Children’s Oncology Group (COG) clinical trial ACNS0831 and underwent multi-agent induction chemotherapy with carboplatin, vincristine, cyclophosphamide, and etoposide. A follow-up MRI 3 months later showed interval progression in the size of the thalamic tumor (Fig. 1b). The patient’s clinical presentation continued to decline with new right leg weakness.

Based on the patient’s poor response, further pathology review was performed at multiple centers, as well as centrally at COG to clarify her diagnosis. Pathology showed fragments of densely cellular glial neoplasm composed of relatively uniform, regularly distributed, ovoid cells with round, centrally located nuclei, stippled chromatin, and clear-to-eosinophilic cytoplasm (Fig. 1c). The background demonstrated a prominent, arborizing (chicken-wire) capillary network, and scattered calcospherites. Vague perivascular structuring was evident. There were more than five mitoses per 10 high-power fields. No necrosis was seen (Fig. 1d). The tumor was variably positive for glial fibrillary acidic protein (GFAP) and oligodendrocyte transcription factor (Olig2) and negative for all neuronal markers. The tumor was negative for isocitrate dehydrogenase 1 (*IDH1*) mutation, *KIAA:BRAF* fusion, and *BRAF v600E* mutation. Additionally, the tumor was positive for loss of 1p, but negative for co-deletion of 19q. At this point, a diagnosis of “anaplastic glioma with clear cell features” was rendered with the comment that these features were consistent with the aggressive entity often diagnosed as central or thalamic oligodendroglioma in the pediatric population. The patient was treated with conformal fractionated external beam radiation therapy (RT) for 6 weeks. During RT, the patient developed moderate headaches that were treated effectively with dexamethasone. At the end of this treatment, the patient had some improvement in her clinical exam and a post-RT MRI showed a partial response.

Due to the rarity and poor prognosis of this diagnosis, the patient was enrolled on PEDS-MIONCOSEQ, which was approved by the University of Michigan’s Institutional Review Board. PEDS-MIONCOSEQ is a precision oncology study involving whole-exome (paired tumor and germline DNA) and transcriptome (tumor RNA) sequencing and genetic counseling. Clinically integrated sequencing was performed according to previous published methodology.^16^ Nucleic acid preparation, high-throughput sequencing, and computational analysis were performed using standard protocols in our sequencing laboratory in the Michigan Center for Translational Pathology, which adheres to the Clinical Laboratory Improvement Amendments (CLIA).

Sequencing confirmed 1p loss on copy number analysis and revealed an *FGFR3* copy gain with an additional activating in-frame fusion of *FGFR3* with phosphoglycerate dehydrogenase (*PHGDH*) and increased *FGFR3* expression (see supplementary file for sequencing methods). In addition, a recurrent *FBXW7* (p.R465H) mutation was seen that has been described before but was of unclear significance in this case.^17, 18^ The activating in-frame fusion of *FGFR3-PHGDH* is a canonical *FGFR3* fusion.^19^ The fusion occurs at the very 3’ end of *FGFR3*, with the extracellular domain, transmembrane domain and kinase domains intact (Fig. 1e, f). Sequencing also revealed homozygous deletion of cyclin-dependent kinase (CDK) 11A and CDK11B, as well as a number of other copy gains and copy losses (Fig. 1f). At this time, we were unaware of any clinical significance of these additional mutations. The FGFR3 fusion results were discussed in the teleconferenced University of Michigan Brain Tumor Precision Medicine Conference. In this discussion, it was felt that the activating *FDFR3-PHGDH* gene fusion and increased *FGFR3* gene expression were likely the primary tumor driver. Due to the overall poor prognosis and lack of established effective adjuvant therapy for pediatric anaplastic thalamic oligodendroglioma, recommendation was made to pursue experimental therapy targeted to the patient’s *FGFR* fusion. The group reviewed clinically relevant FGFR inhibitors and their likelihood of blood brain barrier (BBB) penetration, and confirmed that no relevant clinical trials were available.

**Fig. 2:**
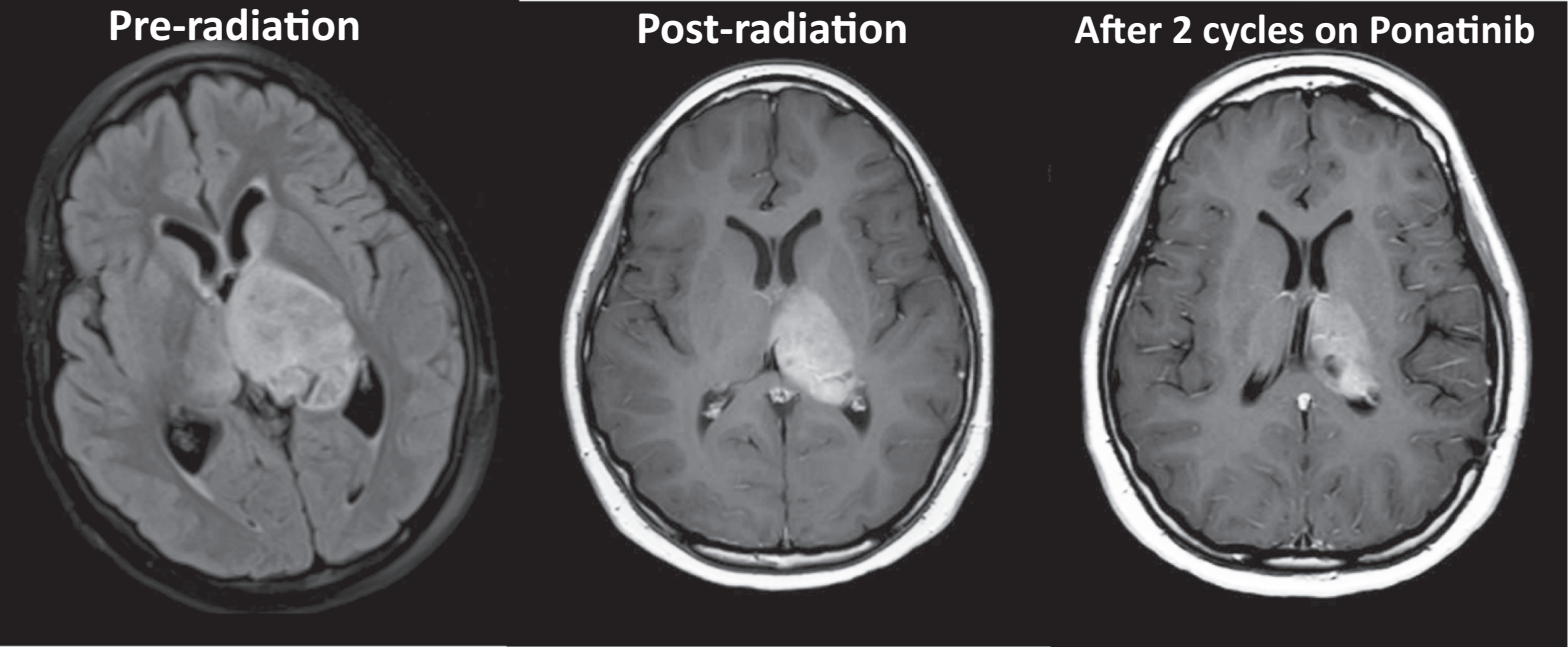
Axial T2 FLAIR image of the thalamic tumor pre-radiation (left), post-radiation (November 2016, center) and after two cycles of ponatinib (March 2017, right). Dimensions of the post-radiation tumor are 45.6 mm × 28.6 mm × 35.0 mm compared to 40.3 mm × 26.6 mm 36.0 mm after two cycles on ponatinib. This represents a 15% reduction in volume

Due to the promising preclinical data suggesting effective in vitro and in vivo activity against glioblastoma cells and the promising characteristics of ponatinib to penetrate the central nervous system (CNS), as well a case report describing ponatinib use in a pediatric patient, we ultimately decided to initiate ponatinib for this patient.^20–23^ The patient weighed 75 kg and was initially started on a treatment regimen of 15 mg of ponatinib daily, with a goal dose of 45 mg orally. After 2 months, the patient’s dose was increased to 30 mg daily for the second cycle. After two cycles, the patient had demonstrated a partial response (15% reduction compared to initial post-radiation scans, Fig. 2). While response attribution between prior radiation and targeted therapy is difficult to fully ascertain (most of her response occurred in response to radiation), this response has persisted for 7 months of therapy at the time of writing this paper. The patient had experienced minimal side effects other than an increase in headache, which are well controlled. Response will continue to be assessed by determining if the patient shows reduction in tumor size on surveillance brain imaging, no new lesions, and stable or improving clinical symptoms.

## Discussion

Gliomas are the most common malignant human brain tumors, and high-grade glioma (HGG) encompasses those with the worst prognosis, including anaplastic oligodendrogliomas (WHO grade III), anaplastic astrocytomas (WHO grade III) and glioblastomas (WHO grade VI).^24^ Even with current treatment modalities, the prognosis for pediatric HGGs remains poor.^25–27^ Fortunately, HGG are relatively rare, comprising less than 20% of pediatric brain tumors.^28^ The median survival for pediatric patients with HGG is less than 2 years.^29^ Oligodendrogliomas comprise about 9% of all gliomas and are classically classified in adults as a glioma harboring a 1p/19q co-deletion.^30^ In adult glioma, 1p/19q co-deletion frequently co-occurs with a mutation in IDH1.^31^ Recent studies have distinguished clinical and molecular sub-types of grade II–IV glioma based on the status of IDH1 mutation, 1p/19q co-deletion, and telomerase reverse transcriptase (TERT) promoter mutations.^31, 32^ While great strides have been made to identify consistent molecular markers in adult oligodendrogliomas, it is widely recognized that pediatric oligodendrogliomas have a vastly different molecular make-up.^8–11^ The current study supports other molecular data, which demonstrates that partial loss or alteration of 1p is common in pediatric central oligodendrogliomas.^3^ This finding is unique compared to hemispheric pediatric oligodendrogliomas, which rarely demonstrate partial loss or alteration of 1p.^10, 33, 34^ Thus, it is imperative to discover new molecular markers to guide the treatment of pediatric central oligodendrogliomas.

Molecular analysis of our patient’s tumor demonstrated an *FGFR3* copy gain with an activating in-frame fusion of *FGFR3-PHGDH*. The critical signaling domains of FGFR remain intact, allowing for the potential utilization of FGFR inhibitors against this fusion. Gene rearrangements of *FGFR* have been associated with cholangiocarcinoma, breast cancer, bladder cancer, thyroid cancer, and prostate cancer.^19, 35^ In addition, a sporadic *FGFR1* mutation has been observed in two medulloblastomas, one low-grade glioma, and three glioblastoma cases.^36^ FGFR inhibitors were demonstrated to be effective in the clinical treatment of tumors with *FGFR* mutations.^19^

Interestingly, PHGDH initiates a pathway that allows for the de novo synthesis of serine and has been recognized in an oncogenic and prognostic role in breast cancer, melanoma, cervical cancer, non-small cell lung cancer, and colon cancer.^37^ PHGDH has also been shown to induce proliferation and invasion in glioma cells.^38^ The molecular configuration of PHGDH has been described to primarily be composed of two substrate-binding domains, an NAD^+^/NADP^+^-binding domain, and a regulatory domain.^39^ The fusion we report would only leave a portion of the regulatory domain intact. No previous reports of an *FGFR3-PHGDH* fusion have been reported; however, our finding strengthens the role of *PHGDH* as a relevant tumor driver in human gliomas.

This patient was a poor candidate for surgical resection of the tumor given that the tumor had infiltrated the patient’s bilateral thalami. This, in combination with the patient’s molecular findings, caused us to consider targeted therapies. Given that the patient’s tumor demonstrated increased *FGFR3* expression, the *FGFR* tyrosine kinase pathway was deemed as the likely tumor driver, encouraging our team to implement FGFR inhibitor therapy with ponatinib.

Ponatinib is a tyrosine kinase inhibitor (TKI) that was initially approved to treat patients with chronic myelogenous leukemia (CML) and Philadelphia chromosome-positive acute lymphoblastic leukemia (ALL) that were resistant to BCR/ABL TKIs.^40^ Ponatinib has also demonstrated potent inhibitory activity against *FGFR1-4* with an IC_50_ of <40 nM.^41^ While ponatinib has not been tested in oligodendrogliomas, it has been shown to have in vitro activity against glioblastoma cells and has caused tumor reduction and apoptosis in a murine xenograft glioblasoma model.^23^ Importantly, in a murine model, ponatinib demonstrated excellent CNS penetration with a brain to plasma concentration ratio of 0.88.^22^ The ability of ponatinib to cross the BBB and achieve levels that are above in vitro IC_50_ levels ultimately influenced the decision to pursue treatment with ponatinib rather than pazopanib.^22, 42^ Recently, a publication raised the question of CNS penetration in a case report of an adult with CNS-positive leukemia who failed to respond to ponatinib.^21^ As per our knowledge, this is the first published use of ponatinib for a primary CNS tumor. Future studies are warranted to further clarify its role in patients with brain tumors harboring FGFR alterations.

In conclusion, we report molecular data for a case of pediatric central oligodendoglioma with a fusion of *FGFR3-PHGDH* and our rationale for pursing personalized, target therapy for the patient’s tumor with ponatinib. Our work demonstrates the benefit of pursuing clinically integrated sequencing in high-risk pediatric brain tumors, strengthens the role of *PHGDH* as a relevant driver of human glioma, and reports a *FGFR3*-containing fusion that may play a role in similar cases.

### Ethical approval

Consent was obtained from the family of the patient at the time of enrollment for the sequencing of her tumor. The methods were performed in accordance with relevant guidelines and regulations and approved by the University of Michigan’s Institutional Review Board.

### Data availability

All relevant data are available from the authors.

## Electronic supplementary material

Supplementary Materials

## Electronic supplementary material

**Supplementary information** accompanies the paper on the *npj Precision Oncology* website (10.1038/s41698-017-0036-8).
