# Author Correction: Identification and targeting of an FGFR fusion in a pediatric thalamic “central oligodendroglioma”

**DOI:** 10.1038/s41698-018-0054-1

**Published:** 2018-04-25

**Authors:** Joseph R. Linzey, Bernard Marini, Kathryn McFadden, Adonis Lorenzana, Rajen Mody, Patricia L. Robertson, Carl Koschmann

**Affiliations:** 1 Department of Pediatrics, Division of Pediatric Hematology/Oncology, University of Michigan Medical School, Ann Arbor, MI 48109 USA; 2 Department of Pharmacy Services, University of Michigan Medical School, Ann Arbor, MI 48109 USA; 3 Department of Pathology, University of Michigan Medical School, Ann Arbor, MI 48109 USA; 4 Department of Pediatric Hematology-Oncology, St. John’s Hospital Grosse Pointe Woods, Detroit, MI 48236 USA; 5 Department of Pediatrics, Division of Neurology, University of Michigan Medical School, Ann Arbor, MI 48109 USA

**Correction to**: *npj Precision Oncology* **1**:29; 10.1038/s41698-017-0036-8, Published online: 07 September 2017

In the original version of this Article the Supplementary Information was missing. This file has now been added to the HTML version of this Article.

