# Supplementary material for: Identification and targeting of an FGFR fusion in a pediatric thalamic “central oligodendroglioma”
Source: NPJ Precis Oncol. 2017 Sep 7;1:29. doi: 10.1038/s41698-017-0036-8 (PMC5871816; doi:10.1038/s41698-017-0036-8)
Supplement: Supplementary file 1 — Supplementary Materials [file 41698_2017_36_MOESM1_ESM.docx]

CLINICALLY INTEGRATED SEQUENCING

Clinically integrated sequencing was performed according to previous published methodology.^1-3^ The Pediatric Mi-Oncoseq Study was initiated in May 2012 and continues as of February 2018. All patients or their parents or legal guardians provided informed consent (written assent if >10 years) and received mandatory pre-enrollment genetic counseling regarding the potential risks of incidental genetic findings. Once enrolled, a patient’s clinical course was captured quarterly in order to document clinical status and treatment decisions made by the primary team since the last follow-up. Briefly, a board-certified neuro-pathologist (K.Mc) evaluated histologic sections for estimation of tumor content before submitting tissue for sequencing (minimum of 70% tumor content required for sequencing). Nucleic acid preparation and high-throughput sequencing were performed using standard protocols in our sequencing laboratory, which adheres to the Clinical Laboratory Improvement Amendments (CLIA).^1-3^ Paired-end whole-exome or Onco1700 libraries from tumor samples that were matched with normal DNA and with transcriptome libraries either from polyadenylated tumor RNA (PolyA + transcriptome) or from total RNA captured by human all-exon probes (capture transcriptome) were prepared and sequenced using the Illumina HiSeq 2000 and 2500 (Illumina Inc). Aligned exome and transcriptome sequences were analyzed to detect putative somatic mutations, insertions and deletions (indels), copy-number alterations, gene fusions, and gene expression as described previously.^1-3^

As previously described,^[1-3](#_ENREF_13)^ pathogenicity of germline variants was determined through a review of the published literature, public databases including but not limited to ClinVar, the Human Genome Mutation Database, the Leiden Open Variation Databases, and variant specific databases (eg, International Agency for Research on Cancer TP53 Database, International Society for Gastrointestinal Hereditary Tumors mutation databases). Only variants that had been previously described as pathogenic were considered for disclosure. Variants with conflicting pathogenicity reports and variants not previously reported were considered to be of uncertain significance and were not considered for disclosure. Following disclosure, familial testing was recommended. Clinical relevance of somatic variants was investigated using an integrated approach incorporating technical considerations, (eg, recurrence, variant allele fraction, expression levels, and predictive algorithms for pathogenicity), variant specific information (ie, ClinVar, published literature, and curated gene specific resources), as well as published correlations of drug and variant sensitivity profiles. Considerations of tumor heterogeneity, including clonal vs sub-clonal mutation were addressed by comparing variant allele fractions and copy-number estimates for each of the mutations to post-sequencing estimates of tumor content derived from single-nucleotide variation and copy-number analyses. Alterations of less than 5% allele fraction were judged to be sub-clonal and not used as basis for therapeutic clinical action. Each of the aberrations for which clinical action was based in this study were judged to be clonal.

**Supplementary Text References**

1. Mody RJ, Wu YM, Lonigro RJ, et al. Integrative Clinical Sequencing in the Management of Refractory or Relapsed Cancer in Youth. *Jama.* 2015;314(9):913-925.

2. Robinson DR, Wu YM, Vats P, et al. Activating ESR1 mutations in hormone-resistant metastatic breast cancer. *Nature genetics.* 2013;45(12):1446-1451.

3. Wu YM, Su F, Kalyana-Sundaram S, et al. Identification of targetable FGFR gene fusions in diverse cancers. *Cancer discovery.* 2013;3(6):636-647.
